# The *C–S–A* gene system regulates hull pigmentation and reveals evolution of anthocyanin biosynthesis pathway in rice

**DOI:** 10.1093/jxb/ery001

**Published:** 2018-01-18

**Authors:** Xingming Sun, Zhanying Zhang, Chao Chen, Wei Wu, Nannan Ren, Conghui Jiang, Jianping Yu, Yan Zhao, Xiaoming Zheng, Qingwen Yang, Hongliang Zhang, Jinjie Li, Zichao Li

**Affiliations:** 1 Key Laboratory of Crop Heterosis and Utilization, Ministry of Education/Beijing Key Laboratory of Crop Genetic Improvement, Department of Plant Genetics and Breeding, China Agricultural University, Beijing, China; 2 Institute of Crop Science, Chinese Academy of Agricultural Sciences, Beijing, China

**Keywords:** Color, domestication, evolution, flavonoid, gene interaction, gene network, rice

## Abstract

Floral organs in rice (*Oryza sativa*) can be purple, brown, or red in color due to the accumulation of flavonoids, but the molecular mechanism underlying specific organ pigmentation is not clear. Here, we propose a *C–S–A* gene model for rice hull pigmentation and characterize it through genetic, molecular, and metabolomic approaches. Furthermore, we conducted phylogenetic studies to reveal the evolution of rice color. In this gene system, *C1* encodes a R2R3-MYB transcription factor and acts as a color-producing gene, and *S1* encodes a bHLH protein that functions in a tissue-specific manner. C1 interacts with S1 and activates expression of *A1*, which encodes a dihydroflavonol reductase. As a consequence, the hull is purple where functional *A1* participation leads to high accumulation of cyanidin 3-*O*-glucoside. Loss of function of *A1* leads to a brown hull color due to accumulation of flavonoids such as hesperetin 5-*O*-glucoside, rutin, and delphinidin 3-*O*-rutinoside. This shows a different evolutionary pathway of rice color in *japonica* and *indica*, supporting independent origin of cultivars in each subspecies. Our findings provide a complete perspective on the gene regulation network of rice color formation and supply the theoretical basis for extended application of this beneficial trait.

## Teaser

The *C–S–A* gene system determines rice hull pigmentation. A conserved color-producing model reveals the independent origin and evolution of the anthocyanin biosynthesis pathway in rice.

## Introduction

As major secondary metabolites, flavonoids are well known for giving distinctive floral organ colors and as antioxidants with beneficial effects on human health (Mol *et al.*, 1998; Seyoum *et al.*, 2006; Jennings *et al.*, 2012; Wedick *et al.*, 2012; Cassidy *et al.*, 2013). The flavonoids are classified into six main groups, namely chalcones, flavanones, flavones, flavonols, anthocyanins, and proanthocyanidins. Their formation is catalysed by enzymes encoded by structural genes utilizing phenylalanine as a common substrate (Winkel-Shirley, 2001). A conserved MBW (MYB–bHLH–WD40) complex is believed to regulate the common pathway of flavonoid biosynthesis, especially for anthocyanins and proanthocyanidins, but flavonol biosynthesis seems to be regulated only by MYB transcription factors (Hichri *et al.*, 2011; Xu *et al.*, 2015).

In maize, a purple or brick-red color in specific tissues is determined by the accumulation of anthocyanin or phlobaphene, respectively (Styles and Ceska, 1975, 1977). Anthocyanin biosynthesis is coordinately regulated by the *C1*/*P1* and *R*/*B* genes, which encode R2R3-MYB and bHLH transcription factors, respectively (Dooner *et al.*, 1991). Phlobaphene biosynthesis is activated by the *P1* (MYB) gene alone (Grotewold *et al.*, 1994). In addition to the different colors in floral organs, anthocyanin also occurs in various vegetative tissues in the maize plant. The tissue-specific pigmentation patterns are regulated by members of the *R* gene family, which includes *R*, *B*, *Lc*, and *Sn* genes (Ludwig and Wessler, 1990).

Although most modern rice cultivars have no flavonoid pigmentation, there are a few varieties that possess purple, brown or red color, in floral organs (see Supplementary Fig. S1A at *JXB* online). Purple color also occurs in leaves, leaf sheaths, internodes, and ligules. A genic model suggested that coloration depended on three different kinds of genes, namely a color-producing gene, an activator gene for anthocyanin biosynthesis, and a tissue-specific pigmentation gene (Nagao *et al.*, 1962; Takahashi, 1982). The potential nutritional value of colored pericarp has attracted attention from geneticists and breeders. Purple pericarp (or black rice) accumulates anthocyanin and is predicted to be controlled by alleles at three loci, named *Kala1*, *Kala3* and *Kala4* (Reddy *et al.*, 1995; Maeda *et al.*, 2014). A rearrangement in the promoter region of *Kala4* is responsible for determination of the purple or black pericarp (Oikawa *et al.*, 2015). Red pericarp is enriched in proanthocyanidin and is controlled by *Rc* (bHLH) with functional *Rd* (dihydroflavonol reductase), whereas brown pericarp is formed without *Rd* participation (Sweeney *et al.*, 2006; Furukawa *et al.*, 2007). Apart from the pericarp, the genetic patterns and genes responsible for the coloration in other organs are unclear. Although earlier work determined that *OsC1* acts as a chromogen gene and functions in various organs such as the apiculus and leaf sheath, its function at the molecular level has not been clarified (Saitoh *et al.*, 2004; Gao *et al.*, 2011). It was also suggested that the *pl* locus containing two adjacent genes was responsible for leaf coloration (Sakamoto *et al.*, 2001). But, until now, there has been no systematic understanding of the gene regulation network in the coloration of specific organs.

Wild rice (*Oryza rufipogon*) has various colors such as black hull, red pericarp, purple awn, and purple leaf margin (Li and Chen, 1993), but most cultivars have lost the color in these organs through long-term artificial selection and improvement. This is of great significance for understanding the origin and evolution of rice through investigation of the genes controlling color formation. The apiculus, as the remnant of the awn, maintains its color in some cultivars and seems not to have undergone artificial selection during domestication (Saitoh *et al.*, 2004; Choudhury *et al.*, 2014). Hence, a good insight can be had into coloration in cultivars by evaluating sequence variations of the related genes underlying apiculus coloration. In addition, most evolution studies focus on individual genes, but the phenotypes are determined by metabolic pathways and their regulatory cascades. Thus understanding phenotypic diversiﬁcation requires the study of metabolic pathway evolution.

In this study, by genetically dissecting hull color, we illustrate the *C–S–A* gene system in the regulation of hull pigmentation. The independent evolution of rice color is outlined by phylogenetic analysis of the genes in this system.

## Materials and methods

### Rice materials

The near isogenic lines (NILs) for constructing mapping populations were as follows:

(i) PH NIL, a purple hulled NIL of BC_2_F_6_ generation in Nipponbare background. The donor parent was a temperate *japonica* variety, XZM, with purple hulls.
(ii) PA NIL, a purple awned NIL of BC_4_F_8_ generation. It was obtained from the crosses and backcrosses between Nipponbare and a temperate *japonica* with purple awns.

NILs for transformation, expression and/or metabolite detection studies were as follows:

HC1, a cSa-type NIL selected from recessive plants in F_4_ population I-2. It has straw-white hulls caused by non-functional *C1* and *A1* alleles.
HC2, a CSA-type NIL selected from the dominant segregates in F_4_ population I-1. It has purple hulls caused by functional *C1*, *S1*, and *A1* alleles.
HC3, a CSa-type NIL derived from recessive segregates of the line F_4_-III. It has brown hulls caused by functional *C1* and *S1* with non-functional *A1* alleles.
HC4, a cSA-type NIL selected from the recessive segregates in F_4_ population Ⅰ-1. It has straw-white hulls caused by functional *S1* and *A1* with non-functional *C1* alleles.
HC5, a CsA-type NIL with BC_3_F_6_ generation selected from recessive plants of the line F_4_-II. It has purple apiculus caused by functional *C1* and *A1* with non-functional *S1* allele.

### Rice germplasm for gene sequencing and evolutionary analysis

Cultivars used for haplotype analysis, neutrality tests and phylogenetic analyses were collected from the 3K-rice project (Rice Functional Genomics and Breeding database, RFGB). The genome sequences of *C1* and *A1* of 108 wild rice varieties were supplied by Q. W. Yang.

All rice plants were grown under natural conditions in paddy ﬁelds located in Beijing or Sanya in Hainan province. Detailed information for accessions is provided in Supplementary Table S1.

### Plasmid construction and rice transformation

Overexpression vectors were constructed by amplifying the *C1* (*Os06g0205100*) from genomic DNA and the *S1* (*Os04g0557500*) coding DNA sequence (CDS) from cDNA of PH NIL; the PCR products were digested with *Bam*HI and *Spe*I, followed by cloning into the binary vector pMDC32 (Curtis and Grossniklaus, 2003). To generate the *C1* complementation plasmid, a fragment of about 4.9 kb DNA containing 2.7 kb promoter, 1.4 kb coding region and a 0.8 kb 3′-untranslated region (UTR) of *C1* was amplified from DNA of PH NIL, with digestion by *Pme*I and *Asc*I, and cloned into the pMDC83 vector (Curtis and Grossniklaus, 2003). For the complementation test of the *A1* gene, a 4.5 kb DNA fragment containing 2.1 kb promoter, 1.6 kb coding region and a 0.8 kb 3′-UTR was amplified from DNA of PH NIL, digested with *Pac*I and *Asc*I, and cloned into pMDC162 (Curtis and Grossniklaus, 2003). To construct the *C1* RNAi vector, a 364 bp fragment from the third exon of *C1* was amplified from the DNA of Nipponbare and linked into pTCK303 (Wang *et al.*, 2004).

All constructed plasmids were introduced into *Agrobacterium tumefaciens* strain EHA105 for infection. Rice transformation was performed as described (Hiei *et al.*, 1994). Primers used for fine mapping, gene sequencing, RT-PCR, and vector construction are shown in Supplementary Table S2.

### RNA extraction and quantitative RT-PCR

Total RNA was extracted from various rice tissues using RNAiso Plus (Takara). cDNA was generated in 25 μl reaction mixtures containing 2 μg DNase I-treated RNA, 200 U M-MLV reverse transcriptase (Takara), 40 U Recombinant RNase Inhibitor (Takara) and 0.1 μΜ oligo (dT)_18_ primer. RT-PCR was performed in total volumes of 10 μl containing 5 μl SYBR premix EX Taq (Takara), 0.2 μl Rox Reference Dye II (Takara), 0.4 mΜ gene-specific primers and 0.5 μl cDNA on an ABI 7500 real time PCR system (Applied Biosystems). The ubiquitin gene *Os03g0234200* was used as an internal reference.

### Yeast two-hybrid assay

Intact or a series of truncated CDSs of *C1* was amplified and subcloned into the pGADT7 vector (Takara) between *Eco*RI and *Bam*HI sites. Intact *S1* or its truncated CDS was fused to the pGBKT7 vector between the *Eco*RI and *Bam*HI sites. Transformed yeast cells were grown on SD−Trp/−Leu (−LT) or SD−Trp/−Leu/−His/−Ade(−LTHA) media plus X-gal. Experimental procedures were performed according to the manufacturer’s user manual (Clontech). Yeast strain AH109 was used in this assay.

### Transient activation assay

Promoters of the gene *CHS* (*Os11g0530600*), *CHI* (*Os03g0819600*), *F3H* (*Os04g0662600*), *F3′H* (*Os10g0320100*), *ANS* (*Os01g0372500*), and *A1* were cloned and fused to the 5′ end of the β-glucuronidase (GUS) gene in the pMDC163 vector as reporters (Supplementary Fig. S2). The function of these genes in anthocyanin biosynthesis was confirmed in Arabidopsis (Shih *et al.*, 2008). *C1* and *S1* were activated by the CaMV35S promoter using the vector pMDC32 (Supplementary Fig. S2). A CaMV35S:LUC plasmid was used as an internal control to estimate the extent of transient expression. GUS/luciferase (LUC) ratios were defined as relative activation activities. Three biological replicates and five technical replicates were assayed for each transformation event.

### Electrophoretic mobility shift assays

The DNA binding domain of C1 or S1 was amplified from PH-NIL and cloned into the pMAL-C5X vector (New England Bio Labs, N8108S) using the Seamless Assembly Cloning Kit (Clone Smarter, C5891). MBP and MBP–C1^1–118^ or MBP–S1^336–451^ fusion proteins were purified using amylose resin (New England Bio Labs, E8021S). DNA probes were amplified and labeled with biotin (Invitrogen/Thermo Fisher Scientific). DNA gel shift assays were conducted according to the manual for the LightShift Chemiluminescent EMSA Kit (Thermo Fisher Scientific, 20148).

### Flavonoid-targeted metabolome analysis

Fully mature seeds were harvested from the field and dehulled to collect the hull samples. Each colored hull sample was derived from five individual plants and mixed equally. The vacuum freeze-dried hulls were ground using a mixer mill (MM400, Retsch) for 1.5 min at 30 Hz; 100 mg powdered hull was extracted overnight at 4 °C with 1.0 ml 70% aqueous methanol containing 0.1 mg l^−1^ lidocaine (as internal standard). Samples were shaken three times during this period to improve extraction efficiency. After centrifugation for 10 min at 10 000 *g*, the supernatant was filtered through a 0.22 μm syringe filter and then stored at −80 °C. Each colored hull was detected and assayed in three replications.

The sample extracts were analysed using a liquid chromatography (LC)–electrospray ionization (ESI)–tandem mass spectrometry (MS/MS) system (UPLC, Shim-pack UFLC Shimadzu CBM20A system; MS/MS, Applied Biosystems 4500 QTRAP). Analytical conditions were as follows, HPLC: column, Waters ACQUITY UPLC HSS T3 C18 (particle size 1.8 μm, length 2.1 mm×100 mm); solvent system, water (0.04% acetic acid): acetonitrile (0.04% acetic acid); gradient program, 100:0 v/v at 0 min, 5:95 v/v at 11.0 min, 5:95 v/v at 12.0 min, 95:5 v/v at 12.1 min, 95:5 v/v at 15.0 min; flow rate, 0.4 ml min^−1^; temperature, 40 °C; injection volume, 5 μl. The effluent was alternatively connected to an ESI-triple quadrupole-linear ion trap (QTRAP)–MS. Quantiﬁcation of metabolites was carried out using a scheduled multiple reaction monitoring method (Chen *et al.*, 2013).

Relative signal intensities of metabolites were normalized by ﬁrst dividing them by the intensities of the internal standard (lidocaine) and then log2 transforming to improve normality. Principal component analysis was carried out by SIMCA-P software. Differences in the metabolites between three types of colored hulls were determined by two criteria: (i) variable influence on projection (VIP) values obtained from the orthogonal partial least squares discrimination analysis (OPLS-DA) model (VIP≥1), and (ii) fold change ≥2 or fold change ≤0.5 between pairwise comparisons. After conditional selection, 49 flavonoids were used for hierarchical clustering analysis by R (www.r-project.org/) to visibly display the specific accumulation of flavonoids and variations among purple, brown, and straw-white hulls.

For quantification of cyanidin 3-*O*-glucoside by UPLC, absorbance spectra were set between 190 and 700 nm, and chromatograms were acquired at 520 nm. Data were analysed using Waters Empower software. Standard substances were purchased from the Sigma-Aldrich.

### Combinational haplotype analysis

A panel of 145 rice accessions (see Supplementary Table S3) was selected from the mini core collection. The hull and apiculus colors were recorded at two growth stages in field trials at Beijing and Sanya, respectively. The *C1* and *A1* genes were directly sequenced by PCR products, and then subjected to genotyping.

### Color diversification paths analysis

To construct an evolutionary tree of rice color, four functional mutations (three indels and one substitution) of *C1* and two functional mutations of *A1* were selected and their genotypes were identified in 471 accessions. The combined haplotypes of 471 accessions were analysed by MEGA 6 software (Tamura *et al.*, 1994), and the mutational steps among haplotypes were assessed by Arlequin version 3.5 (Excoffier and Lischer, 2010). The Arlequin distance matrix output was imported into Hapstar 0.7 to draw the genealogical map for rice color diversification (Teacher and Griffiths, 2011).

### Neutrality test

A large panel of 108 wild rice and 363 cultivar (135 *japonica*, 228 *indica*) accessions was used to evaluate the π and Tajima’s *D* values of the two genes. For *C1*, single nucleotide polymorphisms (SNPs) in a 5 kb (3 kb upstream and 1 kb downstream of the *C1*) region were used for analysis; for *A1*, SNPs in a 6 kb region covered by *A1* were selected. The data were computed by DnaSP 5.10 software (Librado and Rozas, 2009).

### Allele frequency analysis

A total of 342 varieties (143 landraces and 199 improved varieties) were surveyed for the *C1* and *A1* gene. All of the material was genotyped as functional or non-functional alleles by investigating the functional nucleotide polymorphism sites of each gene. The proportions in each group were displayed in pie charts.

### Phylogenetic analysis

Because of the large sequence diversities for *C1* and *A1* in wild rice, haplotypes containing only one variety were also generated in a spanning tree. But for simplification, we only chose haplotypes containing more than two individuals or integrated haplotypes of single individuals located in the main branch as representative haplotypes. Finally, 75 wild rice, 199 *indica* and 127 *japonica* accessions were selected to construct a minimum-spanning tree. A total of 76 polymorphic sites including 22 (18 SNPs and four indels) from the *C1* gene region and 54 (53 SNPs and one indel) from the *A1* gene region were used in the study. The combined *C1* and *A1* haplotypes of 401 varieties were genotyped by MEGA 6 software and used for constructing a neighbor-joining tree. The data were also used to generate a minimum spanning tree as mentioned above.

## Results

### Genetic dissection of hull color

**Fig. 1.**
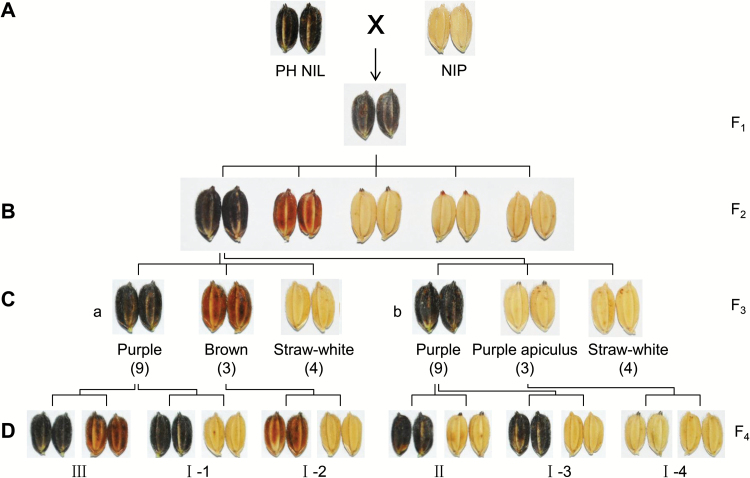
Inheritance of hull coloration. (A) Purple hulled PH NIL was crossed with NIP; the hull color of F_1_ was purple. (B) The F_2_ population segregated into five phenotypes (purple hull, brown hull, purple apiculus, brown apiculus, and straw-white hull). (C) F_3_ populations ‘a’ and ‘b’ segregated in 9:3:4 ratios but with different phenotypes. The line F_3_-a shows the separation of different colors and F_3_-b shows the separation of the pigmentation part. In line F_3_-a, hull color segregated as purple, brown and straw-white. In line F_3_-b, color segregated as purple hull, purple apiculus, and straw-white hull. (D) F_4_ segregating lines were obtained from F_3_ individuals as indicated. Six single gene segregation patterns were confirmed. Lines I-1, -2, -3, and -4 segregated colored (purple or brown) and straw-white phenotype, implying color is controlled by a chromogen gene. Line II segregated in a tissue-specific pattern, implying regulation by a tissue-specific pigmentation gene. The segregation in line III indicated that there is an activator for purple color. The phenotypic data are provided in Supplementary Table S4.

In order to identify genes participating in rice hull pigmentation, we constructed the purple hull NIL, PH NIL, with purple hull derived from XZM in a Nipponbare background (see ‘Materials and methods’). A further backcross was made to Nipponbare to study the inheritance of hull coloration. The F_1_ had purple hulls indicating dominance of this trait (Fig. 1A). The F_2_ population segregated into five distinct types, namely purple hull, brown hull, purple apiculus, brown apiculus, and straw-white hull (Fig. 1B). Under field conditions, purple color could be observed in the apiculus at an early stage when panicles were exposed to sunlight at the initial heading stage. But brown color just began to appear at the wax ripeness stage. Both purple and brown hulls could be easily distinguished at the fully ripened stage (see Supplementary Fig. S1B). Purple and brown color in the apiculus paralleled the hull patterns. Because some individuals did not mature in the crop season, the brown and straw-white hulls were not accurately identified; consequently the exact segregation ratio in the F_2_ population was not obtained. We selected a number of self-pollinated purple hulled plants and examined their F_3_ progeny. Among segregating lines of F_3_, there were two types of 9:3:4 segregation ratios indicating recessive epistatic interactions between two genes. The segregation of the F_3_-a line implied that two related loci determined the different colored hulls, whereas segregation of the F_3_-b line indicated two loci determining specific pigmentation patterns in the hull (Fig. 1C).

We further examined F_4_ populations of F_3_ individuals that had colored phenotype. Among these lines, we identified several populations segregating in a single gene and proposed a genetic model for hull pigmentation. The inheritance patterns of I-1, -2, -3, and -4 indicated there is a color-producing (chromogen) gene because the recessive classes had straw-white hull phenotypes. Line F_4_-II segregated purple hull and purple apiculus in a 3:1 ratio suggesting that the locus involved was specific for hull pigmentation. Finally, the locus responsible for purple *versus* brown in line F_4_-III was likely an activator of anthocyanin biosynthesis (Fig. 1D; Supplementary Table S4), because it is known that purple color is formed by the accumulation of anthocyanin (Reddy *et al.*, 1995). Thus rice hull pigmentation appeared to depend on a chromogen gene, a tissue-specific gene, and an activator gene following previously reported pigmentation patterns (Takahashi, 1982).

### Mapping and candidate gene analyses for hull pigmentation

Firstly, we used recessive individuals obtained from F_4_ lines I-1 and I-2 to map the chromogen genes. The candidate genes were delimited to the same locus between SSR markers RM5754 and RM19565 on chromosome 6 (see Supplementary Fig. S3A). Within this region, the gene *Os06g0205100* encoding a R2R3-MYB transcription factor had been reported as *OsC1* and predicted to be a regulator in the anthocyanin biosynthesis pathway (Saitoh *et al.*, 2004). We compared the nucleotide sequence of this gene between the PH NIL and Nipponbare and found a −GAG deletion in the second exon in Nipponbare that caused loss of a glutamic acid (E) residue in the R3 repeat (Supplementary Fig. S3B). Thus, we considered *Os06g0205100* (*C1*) to be the candidate gene for chromogen.

We similarly mapped the hull-specific pigmentation gene using recessive individuals from F_4_-II and its progeny. The candidate gene was firstly mapped to a 345 kb region between SSR markers RM3820 and MM2687 on chromosome 4. Due to lack of polymorphic markers between the PH NIL and Nipponbare, we generated another mapping population using the PA NIL (straw-white hull with purple awn) crossed with the PH NIL (see Supplementary Fig. S4A). F_1_ plants showed purple hulls, and the hull color of F_2_ individuals segregated 201 purple hull plants: 72 straw-white hulled individuals (χ^2^_3:1_=0.27, *P*>0.05) indicating that a single dominant gene from the PH NIL controlled hull pigmentation. This population was used for fine-mapping of the causal gene. Finally, we mapped the candidate gene to a 50.02 kb interval between SNP markers Snp2 and Snp4. The single gene in this region, *Os04g0557500*, encodes a bHLH transcription factor (Supplementary Fig. S4B). By sequence analysis, we found that *Os04g0557500* spanned a 24 kb genomic region and had a unique structure in Nipponbare with transposon elements in the second and sixth introns (Supplementary Fig. S4C). We found no difference among PH NIL, PA NIL, and Nipponbare in the promoter and cDNA. As the only protein-coding gene in the mapped region, *Os04g0557500*, was presumed to be the candidate gene for hull-specific pigmentation and named as *S1* for organ specificity.

The line F_4_-III and its F_5_ derivatives were selected for mapping the activator gene for anthocyanin biosynthesis. A total of 1268 recessive individuals (brown hulls) were screened by SSR markers, and finally the candidate gene was mapped to a 49 kb region on chromosome 1. In the mapped region, *Os01g0633500* encodes a dihydroflavonol reductase (DFR) predicted to catalyse the conversion of dihydroflavonols to leucoanthocyanidins, a crucial step in the biosynthesis of anthocyanin (see Supplementary Fig. S5A). Sequence comparisons of *Os01g0633500* revealed a single nucleotide change from C to A in the second exon causing a premature stop in the recessive plants and Nipponbare. Besides, other SNPs causing amino acid changes existed in the coding region (Supplementary Fig. S5B). We considered this gene to be the activator gene in anthocyanin biosynthesis and denoted it as *A1*.

F_2_ individuals with heterozygous genotypes at all three loci were further checked for segregation ratios in the F_3_ generation. The summarized data confirmed the triplex gene model for rice hull coloration (see Supplementary Table S4). We named the genetic model for hull pigmentation as the *C–S–A* system (combinations of the *C1*, *S1*, and *A1* genes). All three genes were functional for hull pigmentation in PH NIL, but not in Nipponbare.

### Functional verifications of the *C–S–A* gene system for hull coloration

**Fig. 2.**
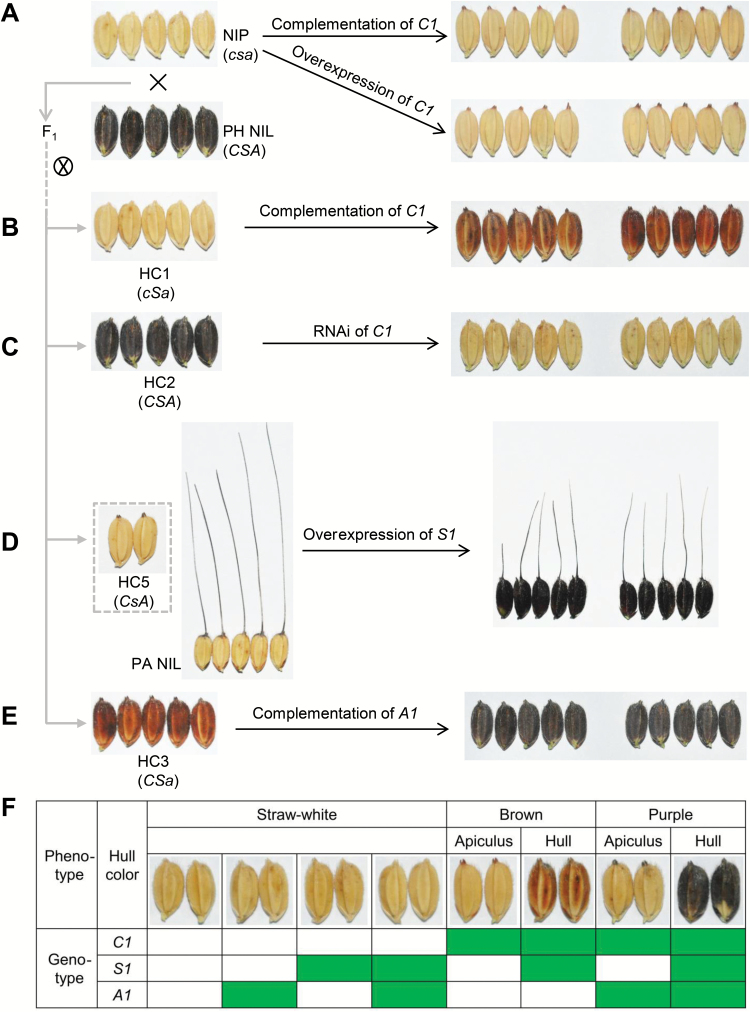
Verification of the *C–S–A* gene system for hull pigmentation. (A) Complementation and overexpression of *C1* in NIP. Both kinds of transgenic plants have brown apiculi. (B) Complementation of *C1* in HC1. Brown hull color was restored in transformants. (C) RNAi of *C1* in HC2. Purple hull color was lost in transformants. (D) Overexpression of the *S1* CDS in PA NIL. The PA NIL has the same pigmentation pattern as HC5. The hull color of transformants changed to purple. (E) Complementation of *A1* in HC3. Transgenic lines restored the purple hull phenotype. (F) Genetic model for hull pigmentation. Green boxes indicate functional alleles; white boxes indicate non-functional alleles. The letters in parentheses denote genotype of *C1*, *S1*, and *A1*, in which upper-case letters represent functional genotypes, and lower-case letters indicate non-functional genotypes. Two independent transgenic lines are shown for each transformation test.

Firstly, the *C1* allele from PH NIL under its native promoter or the cauliflower mosaic virus (CaMV) 35S promoter was transformed into Nipponbare. Both the complementation and overexpression transgenic lines produced brown colors, but it appeared only in the apiculi (Fig. 2A; Supplementary Fig. S6). According to the inheritance pattern of hull coloration, we inferred that *S1* was indispensable for the hull pigmentation, but Nipponbare had a non-functional allele of *S1* and hence all transgenic lines accumulated pigments only in apiculi. Given this, we again introduced *C1* into the HC1. Hull color of all these transformants was brown (Fig. 2B). Further genetic evidence came from RNA interference (RNAi) of *C1* in the HC2; RNAi lines showed loss of purple color in the hull (Fig. 2C; Supplementary Fig. S7A, B). These results confirmed that *C1* was crucial for producing color in rice hulls.

To clarify the function of *S1*, we overexpressed *Os04g0557500* in the PA NIL. Hull color in all transgenic lines was purple (Fig. 2D). Besides, pericarp and leaf blade color were also converted to purple (see Supplementary Fig. S8A–C). Thus, we initially considered that *S1* participated in hull pigmentation and was a determinant of tissue-specific pigmentation.

For validation of the function of *A1* in activation of anthocyanin biosynthesis, we complemented HC3 with functional *A1*. The hull color of all transgenic lines changed from brown to purple (Fig. 2E). However, no color appeared in hulls or other organs when this vector was transferred into Nipponbare (data not shown). This clearly demonstrated that *A1* acted as a catalyst for purple hull coloration and had a role in the *C1*-dependent pathway in rice.

To sum up the *C–S–A* model, *C1* works as a switch in controlling color production; it causes brown color when functioning alone, but in combination with *A1* produces purple color. In addition, purple and brown hulls require a functional *S1*, without which these two colors occur only in the apiculus (Fig. 2F).

### Interaction between C1 and S1 activates structural gene expression

**Fig. 3.**
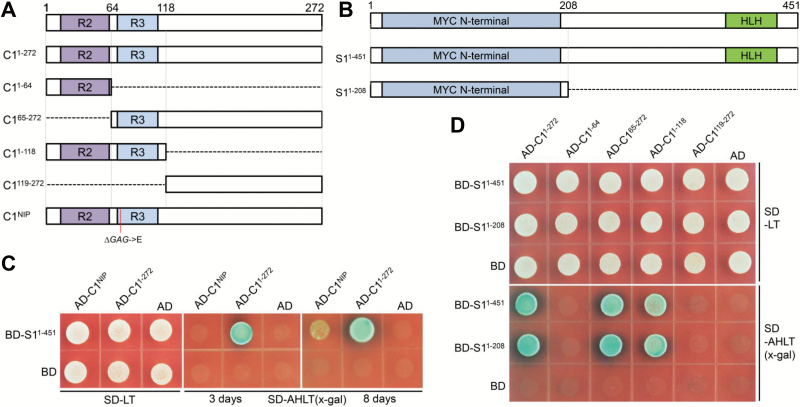
Interactions of C1 and S1 proteins. (A) Schematic representation of the C1 protein domains. Boxes represent different C1 fragments used in yeast two-hybrid assays. C1^NIP^ represents protein from Nipponbare with the deletion of one glutamic acid in the R3 repeat. (B) Schematic representation of the S1 protein domains. (C) Yeast two-hybrid assays for C1 with S1. The pGADT7 and pGBKT7 plasmids contain the GAL4 activation and DNA-binding domains, respectively. C1 with intact protein or protein lacking one amino acid was fused to the pGADT7 plasmid and S1 with intact protein was fused to the pGBKT7 plasmid. The colony growth patterns at 3 and 8 d are shown. (D) Yeast two-hybrid assays for the interactions between truncated C1 and S1 proteins. C1 with intact protein or truncated proteins was fused to the pGADT7 plasmid and S1 with intact protein or its N-terminal domain was fused to the pGBKT7 plasmid.

As *C1* and *S1* together regulated the anthocyanin biosynthesis pathway, we checked the interaction patterns between these two transcription factors by yeast two-hybrid analysis (Fig. 3A, B). The results showed that C1^1–272^ from PH NIL interacted with S1, but C1^NIP^, which lacked a glutamic acid residue in its R3 repeat, had strikingly weakened interaction with the S1 protein (Fig. 3C). We further found that the interaction of C1 and S1 required the amino acids in the 65–118 region of C1 containing the R3 repeat and the amino acids in the 1–208 region of S1 harboring the MYC N-terminal region (Fig. 3D).

**Fig. 4.**
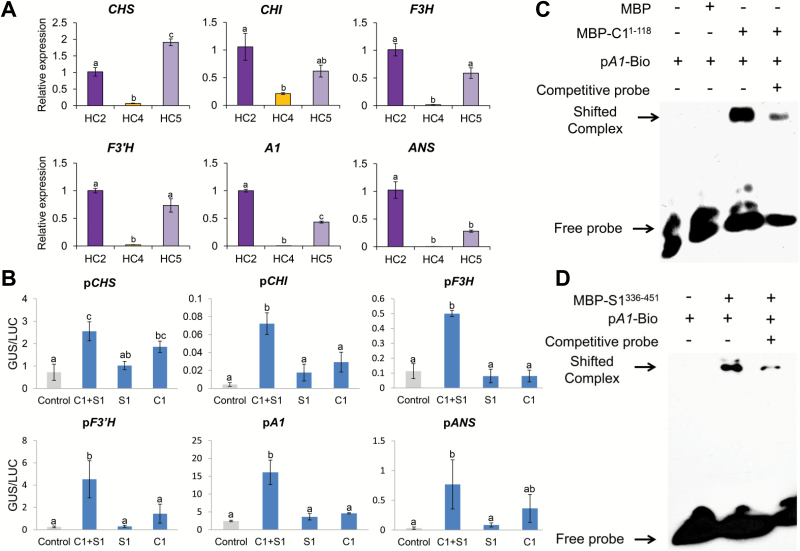
Regulatory patterns of C1 and S1 on structural genes. (A) Expression patterns of the structural genes under *C1* and/or *S1* regulation. Total RNAs were extracted from hulls of HC2 (*CSA*-type NIL, purple hull), HC4 (*cSA*-type NIL, straw-white hull with non-functional *C1*), and HC5 (*CsA*-type NIL, purple apiculus with non-functional *S1*). Error bars indicate±SD (*n*=3). Statistical signiﬁcance was determined by one-way ANOVA. Signiﬁcant differences between means (Duncan, *P*<0.01) are indicated by lower-case letters (a, b, and c) above the bar. (B) Transient activation assays of *C1* and/or *S1* on activating the promoter of structural genes. The reporters and effectors were co-transformed into single tobacco leaves. Relative GUS activities were normalized to the LUC internal control. Error bars represent ±SD (*n*=3). Lower-case letters above the error bars indicate signiﬁcant differences by one-way ANOVA (Duncan, *P*<0.05). (C, D) Binding activity assays of C1 (C) and S1 (D) with *cis*-elements in the *A1* promoter by electrophoretic mobility shift assay.

We next investigated the expression patterns of the structural genes under C1 and/or S1 regulation in the three NILs, HC2, HC4 and HC5. As shown in Fig. 4A, the expression levels of all six structural genes in the anthocyanin branch pathway were significantly decreased in HC4 causing straw-white hull phenotypes. In HC5, the expression of four early biosynthesis genes was maintained at similar levels, but the expression of *A1* and *ANS* (late biosynthesis genes) was significantly decreased relative to those in HC2. We therefore speculate that *C1* plays a more important role than *S1* in activating expression of structural genes in the hulls.

To further confirm activation of structural genes by C1 and/or S1, we performed *trans*-activation assays in *Nicotiana benthamiana*. GUS/LUC ratios indicated that neither C1 nor S1 could separately activate expression of structural genes. However, when C1 and S1 were both expressed in tobacco leaves, GUS activities were strikingly elevated in all six kinds of transformants (Fig. 4B), indicating that the transcriptional complex rather than the individual C1 or S1 factors functioned more efficiently in regulating expression of the structural genes.

To determine whether A1 directly acts downstream of C1 or S1, we conducted electrophoretic mobile shift assays. Both MBP–C1^1–118^ and MBP–S1^336–451^ bound to the DNA motif in the promoter region of *A1 in vitro*, but not MBP alone (Fig. 4C, D), demonstrating that C1 and S1 may regulate expression of *A1* by directly binding to its promoter.

### Exploring unique compounds in each colored hull by flavonoid-targeted profiling

We performed flavonoid-targeted metabolome analyses to determine differences in the quantity and proﬁles of ﬂavonoids among the hulls of HC2 (purple), HC3 (brown), and HC4 (straw-white). A total of 161 flavonoids were detected (see Supplementary Table S5). Principal component analysis of the LC-MS data sets distinguished the metabolite profiles of the three kinds of hulls indicating significant differences in the flavonoid metabolome (see Supplementary Fig. S9).

**Fig. 5.**
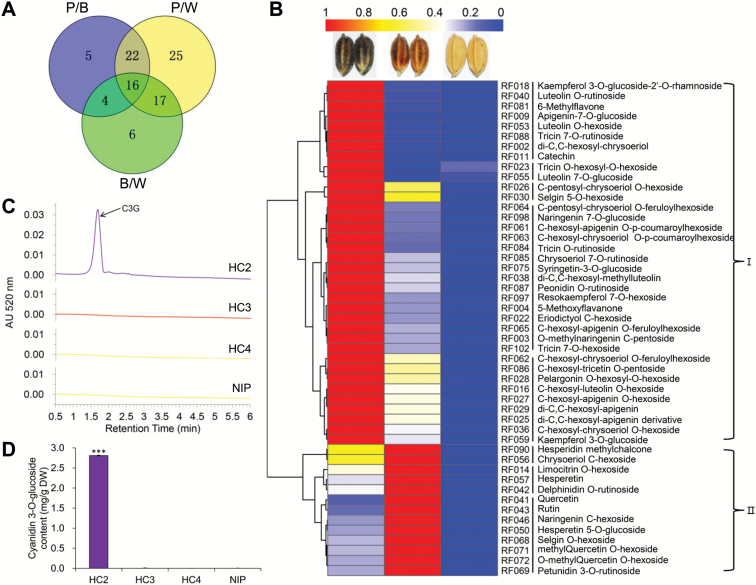
Flavonoid-targeted metabolome analyses of differently colored hulls. (A) Venn diagram showing the numbers of differentially accumulated metabolites in three kinds of colored hulls. P/B indicates differences between purple and brown colored hulls, P/W indicates differences between purple and straw-white hulls, and B/W indicates differences between brown and straw-white hulls. (B) Hierarchical clustering analysis of relative differences of flavonoids in purple, brown, and straw-white hulls. The relative content of each bin was normalized to unit variance and visualized by color. Red indicates high flavonoid abundance; blue indicates low abundance. The flavonoids are listed in Supplementary Table S7. (C) HPLC analysis of anthocyanin content recorded at 520 nm in extracts from hulls of NILs HC2 (*CSA*-type, purple hull), HC3 (*CSa*-type, brown hull), HC4 (*cSA*-type, straw-white hull), and Nip. Cyanidin 3-*O*-glucoside (C3G) showed a peak at 1.68 min. (D) C3G content. ‘DW’, dry weight. Error bars are ±SD (*n*=3). ***P<0.001 (Student’s *t*-test).

By pairwise comparisons of the flavonoid metabolite contents in the three types of hulls, we found the most abundant types of flavonoids were highly accumulated in purple hulls and the lowest levels were in straw-white hulls (Fig. 5A; Supplementary Table S6). Among the 95 flavonoid compounds, we selected those with VIP≥1 in all three comparisons for further analysis.

Finally, 49 flavonoids were selected and their relative contents among purple, brown, and straw-white hulls were used for hierarchical cluster analysis. Based on their specific accumulation patterns in three differently colored hulls, flavonoids were grouped into two clusters. Flavonoids in cluster I accumulated at the highest level in purple hulls whereas those in cluster II accumulated highly in brown hulls (Fig. 5B; Supplementary Table S7). For identifying the major components determining specific color formation, we selected six and five flavonoids with fold changes more than 20 from clusters I and II, respectively. The flavones and proanthocyanidin were specifically representative flavonoids in cluster I (see Supplementary Table S8). Because flavones have no color under natural light conditions, the differential accumulations of these compounds were not the reason for color variation. As reported previously, purple color in rice was produced by accumulation of anthocyanin, mainly cyanidin 3-*O*-glucoside (C3G) (Elsm *et al.*, 2006; Kim *et al.*, 2007; Rahman *et al.*, 2013). Unexpectedly, no kind of anthocyanin was highly accumulated in purple hulls of HC2. We again performed a UPLC assay to specifically quantify the C3G contents in hulls. Purple hulls accumulated C3G at about 2.8 mg g^−1^ dry weight, while brown and straw-white hulls contained none (Fig. 5C, D). In cluster II, the highly accumulated products were flavanones and flavonols. In addition, delphinidin 3-*O*-rutinoside was highly accumulated in brown hulls, at nearly 505-fold higher than in straw-white hulls and 3-fold higher than in purple hulls (Supplementary Table S8). We infer that this specific anthocyanin compound is synthesized by other DFR(s) rather than A1 because of its higher accumulation in both purple and brown hulls. The brown color may be caused by co-pigmentation of delphinidin 3-*O*-rutinoside with specific flavonols and flavanones (Davies and Mazza, 1993; Baranac *et al.*, 1996; Boulton, 2001).

Further analysis revealed that five highly accumulated flavonoids in brown hulls were also present at a similar level in purple hulls; their levels were only 3–10 times less in purple hulls than in brown hulls. This indicated a basic role of *C1* in activating the flavonoid pathway. However, compounds at the highest levels in purple hulls were accumulated at a much lower level in brown hulls and nearly the same as those in straw-white hulls. This indicated a role of *A1* in purple hull coloration with the accumulation of specific flavonoids (Supplementary Table S8).

**Fig. 6.**
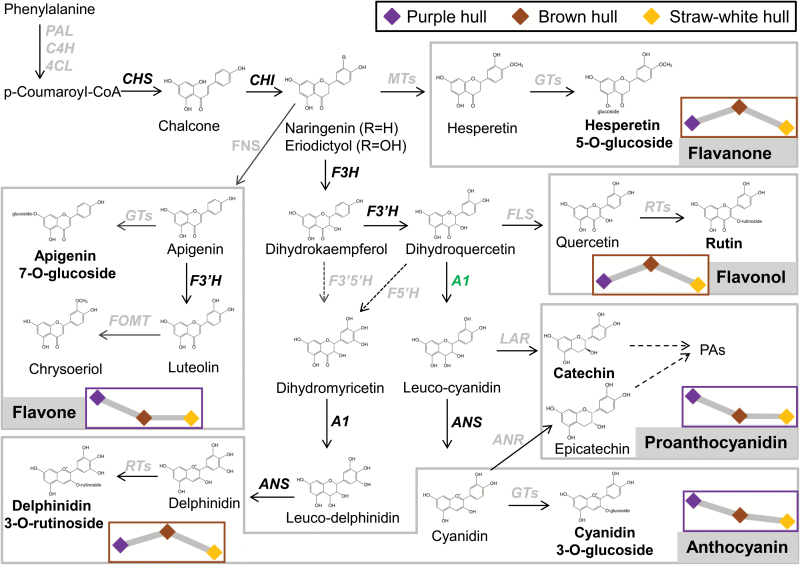
Schematic representation of the flavonoid biosynthesis pathway in rice. Flavonoids were divided into five groups that are indicted by grey shading. Genes encoding the related enzymes are italicized, and genes with well-known function in the flavonoid biosynthesis pathway are shown in bold. The highest accumulations of flavonoids in purple or brown hulls are indicated by purple or brown box borders, respectively. Line charts within boxes indicate the relative flavonoid content in the three kinds of hull.

Taken together, we suggest that flavonoid biosynthesis in rice hulls is initiated by C1 and S1 activation. The purple color is formed by the catalysis of functional A1 and the accumulation of the main products as anthocyanin and proanthocyanidin. If A1 loses its function, the production of flavonoids mainly converts to specific flavonols and flavanones (Fig. 6).

### Correlation of haplotype combinations of *C1* and *A1* with color variations in natural rice germplasm

Since apiculus color is produced by *C1* and *A1*, we could test whether the color-producing model is universal among cultivars by analysing *C1* and *A1* haplotype combinations in natural rice germplasm. The *C1* and *A1* genome sequences of 145 varieties from the mini core collection were surveyed (Zhang *et al.*, 2011). In-depth analysis of *C1* revealed four functional haplotypes (hap1–4) and nine non-functional haplotypes (hap5–13). In the non-functional alleles, three kinds of indels were identified in which 10 bp deletion occurred in almost all *indica* varieties, whereas −TC and −GAG deletions mainly occurred in temperate *japonica* accessions. Besides, a 45 bp substitution between positions 493 and 537 occurred only in temperate *japonica* accessions (see Supplementary Fig. S10A). All of these variations implied that non-function mutations in *C1* arose independently in *indica* and *japonica*. Among *A1* haplotypes, there were nine functional (hap1–9) and three non-functional types (hap10–12). All three non-functional alleles were only in temperate *japonica*, two (hap11 and hap12) of which were newly found as rare variations (Supplementary Fig. S10B).

**Fig. 7.**
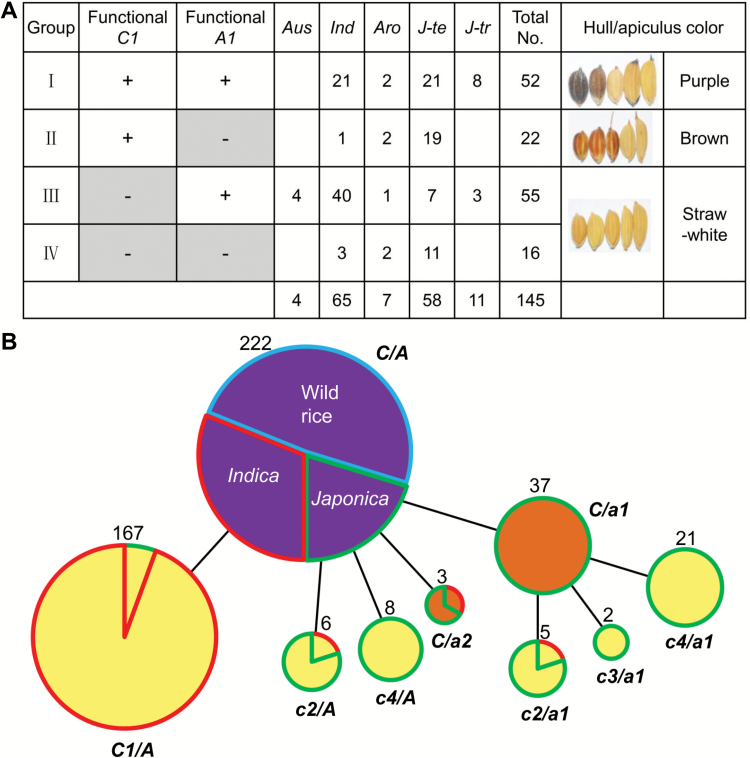
Combinational haplotype analysis of *C1* and *A1* in germplasm reveals the evolution of color. (A) Analysis of haplotype combinations of the *C1* and *A1* genes in lines with hull or apiculus color. One hundred and forty-five varieties were divided into four groups (I–IV) by *C1* and *A1* genotypes. ‘+’ indicates the functional genotype, ‘−’ indicates the non-functional genotype. *Aro*, *aromatic*; *Ind*, *indica*; *J-te*, temperate *japonica*; *J-tr*, tropical *japonica*. Representative phenotypes of varieties in each group are shown. (B) Evolution of rice color. Colors within circles represent the variety color phenotypes. Circle size is proportional to the number of samples with a given haplotype. The number near the circle represents the total number of samples in the group. Colors surrounding circles indicate taxonomic subgroups of varieties: wild rice is blue, *indica* is red, and *japonica* is green. Functional mutations in *C1* are labeled *c1*, *c2*, *c3*, and *c4*, and represent the 10 bp deletion, 2 bp deletion, 3 bp deletion, and sub1, respectively. *a1* and *a2* represent functional mutation sites of hap10 and hap12 in *A1*, respectively.

Subsequently, we investigated the correlation between different haplotype combinations and color phenotypes. The 145 varieties were genotypically divided into four groups by functional or non-functional alleles of *C1* and *A1*. As expected, all accessions in group I had purple hulls or apiculi, and all group II accessions had brown color in either or both tissues, whereas varieties of groups III and IV had no pigments in hull and apiculus (Fig. 7A). These results indicated that the color-producing model controlled by *C1* and *A1* was highly conserved among subspecies. In addition, we found that almost all varieties with brown colors belonged to the temperate *japonica* subgroup.

### Diverse paths of rice color evolution in *indica* and *japonica*

To completely decipher rice color diversification patterns, we first checked the sequence variations in structural genes. Indels and SNPs in coding regions of five structural genes (*CHS*, *CHI*, *F3H*, *F3′H*, and *ANS*) in 471 varieties were surveyed. Nonsense mutations were not found in any of the five genes (see Supplementary Table S9). One indel was present in the 3′ end of *ANS*, but it has been reported that this mutation does not influence ANS function (Reddy *et al.*, 2007). Although non-synonymous mutations were found in all five genes, their genotypes were unrelated to color phenotype. This implies that sequence variations in these five structural genes have no significant role in the evolution of rice color.

We speculated that *C1* and *A1* played determinant roles in the evolution of the anthocyanin metabolic pathway. Genealogical analysis using combined functional mutation sites of *C1* and *A1* revealed different evolutionary patterns of color between *indica* and *japonica* (Fig. 7B). In *indica*, color evolved from purple to straw-white due to the 10 bp deletion in *C1*, but in *japonica* there were at least three ways by which color might have evolved. One way was a change from purple to brown by a functional mutation in *A1*, and then brown changed to straw-white due to a functional mutation in *C1*. The second way was a change from purple to straw-white by a loss-of-function mutation in *C1*. The third way, represented by a few accessions, was from purple to brown color without further change. Since *indica* and *japonica* possessed different functional mutations of *C1* and *A1* in evolving from purple to brown or straw-white, we speculated that the variations occurred after the *indica–japonica* separation.

### Tests of neutrality and effects of selection on *C1* and *A1*

Nucleotide diversity of *C1* in cultivars was higher than in wild rice indicating that *C1* did not undergo selection during domestication. However, we found that colored cultivars underwent balanced selection (Tajima’s *D*=3.13, *P*<0.01) whereas straw-white cultivars had been subject to directional selection (Tajima’s *D*=−1.77, *P*<0.05) (see Supplementary Table S10). Tests on *A1* demonstrated balanced selection across this region in *japonica*. Further analysis of *A1* in *japonica* accessions suggested that this gene had undergone balanced selection in purple-colored varieties and weak directional selection in brown-colored varieties (Supplementary Table S11). In brief, *C1* and *A1* did not undergo directional selection during domestication from wild rice to cultivar, but underwent selection in the diversification process.

**Fig. 8.**
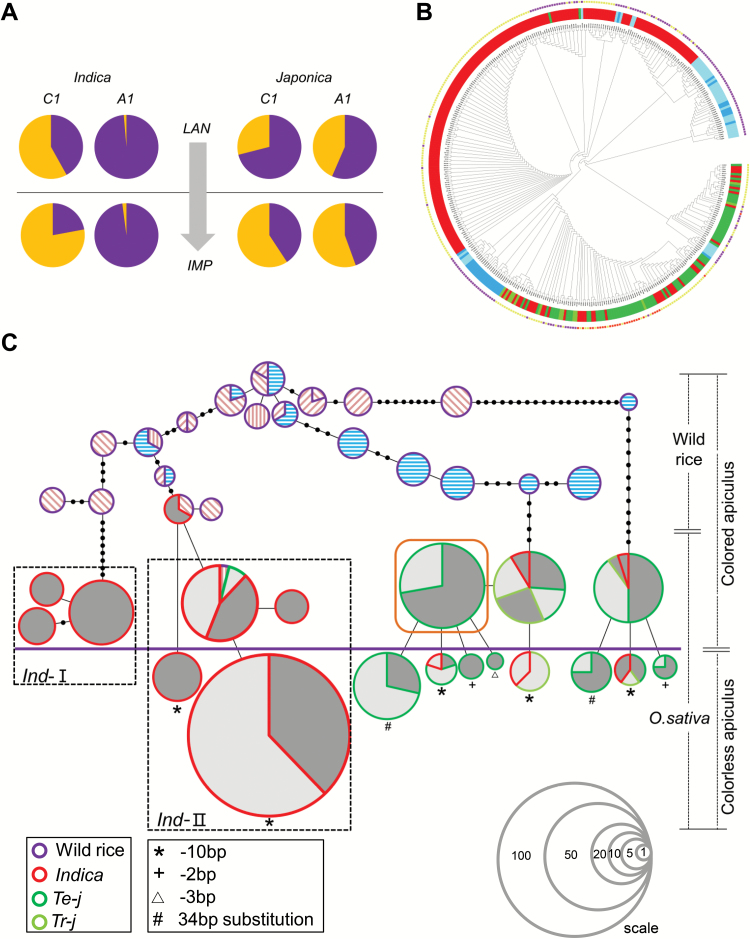
Domestication and diversification of *C1* and *A1.* (A) Allele frequency analysis of *C1* and *A1*. A total 342 rice varieties were identified for functional and non-functional alleles at *C1* and *A1*. In this panel, 143 landraces and 199 improved rice varieties were further grouped as *indica* and *japonica*, respectively. Numbers of functional and non-functional alleles of *C1* or *A1* were used to compute the allele frequency of each gene in four distinct groups. Pie charts above the horizontal line represent landraces whereas improved varieties are shown below the line. (B) Phylogenic tree of rice varieties. The color strip in the inner part of the circle represents the taxonomic subgroup. Dark blue indicates wild rice from China; light-blue represents wild rice outside of China. The colored spots at the outer parts of the tree represent the phenotype of different accessions. (C) A minimum spanning tree of *C1* and *A1* combinations. Circle size is proportioned to the number of accessions with a given haplotype. Black circles on the lines represent mutational steps between alleles. Hatching (for wild rice) within circles indicate the place of origin of the varieties: blue horizontal lines represent accessions from Yangtze and Pearl River valleys; wild rice accessions originating from the Indochinese Peninsula are labeled with pink back-slashes, from South Asia with pink forward slashes, and from the Malay Archipelago with pink vertical lines. Landraces are represented by dark gray and improved rice varieties are represented by pale gray. The thick purple line separates the color phenotypes of varieties; accessions above the line have purple or brown color in their hulls or apiculi, and those below the line have no color. Accessions with brown hulls or apiculi are circled by a brown box. The series of circles located in the lower right corner represent reference scales. Arabic numeral in each circle indicates the number of materials represented by the circle of this size.

We again surveyed the functional allele frequencies of *C1* and *A1* in a panel of 342 accessions. Compared with landrace groups, the functional allele frequencies of *C1* in the improved rice groups were significantly reduced in both *indica* and *japonica*. There was a similar trend for *A1* in *japonica* (Fig. 8A). These results imply that non-functional *C1* and *A1* alleles for straw-white phenotype were selected in the genetic improvement process.

### Independent origins and evolution of rice color

The phylogenetic tree generated by the *C1* and *A1* integrated haplotypes for 401 accessions clearly separated *indica* and *japonica* into different clades along with their recent wild ancestors located in different areas (Fig. 8B).

By creating the haplotype network, the evolutionary lineages of color changes from wild rice to cultivars were clearly revealed (Fig. 8C). It is apparent that distinctly different origins and evolutionary routes occurred in *indica* and *japonica*. All straw-white cultivars arose from colored cultivars and possessed specific non-functional *C1* alleles.

Neither *C1* nor *A1* underwent selection during domestication, and hence the phylogenetic analysis using combined haplotypes of these two genes provided clues for evaluating the evolution of rice coloration, and also provided partial evidence on the origin and evolution of rice species. As shown in the minimum spanning tree (Fig. 8C), the *japonica* originated and was domesticated from *O. rufipogon* in Southern China, but the origin of the *indica* subspecies was unclear. The *indica* was classified into two subgroups (*ind*-I and *ind*-II) and their supposed wild rice ancestors were from Southeast Asia and South China. We assumed that geographically distributed *indica* varieties independently originated from wild rice in their local areas (see Supplementary Fig. S11).

## Discussion

### The *C–S–A* gene system is possibly universal in rice organ coloration except for pericarp

In the *C–S–A* gene system, *C1* and *A1* collectively determine the color variation, whereas *S1* diversifies the pigmented tissues (see Supplementary Fig. S12). Although pigmentation depends on a series of genes belonging to the flavonoid pathway, natural variations that caused functional mutations mainly occurred in these three genes. It seems that this gene system is universal in regulating various organs’ pigmentation. Nonetheless, we cannot exclude the possibility that other R2R3-MYB or bHLH proteins participate in the regulatory network. We collected seven black rice varieties and all had the functional *A1* allele. Three of these varieties had functional *C1* and also possessed purple color in the hull. But in the other four varieties, purple color appeared only in the pericarp, and *C1* in these four varieties showed loss-of-function (Supplementary Table S12). In addition, when *S1* was overexpressed in Nipponbare, the pericarp color became reddish-brown (Supplementary Fig. S13A, B). Therefore, we speculate that *C1* determines color production in most organs except pericarp. The pericarp color is produced by coordinated regulation of *S1* with other MYB-like transcription factors rather than *C1* (Maeda *et al.*, 2014).

### Does WD40 protein participate in anthocyanin biosynthesis in rice?

The MBW complex consists of MYB–bHLH–WD40 transcription factors and is the main regulatory unit for anthocyanin/proanthocyanidins biosynthesis in plants (Hichri *et al.*, 2011). In Arabidopsis, *TTG1* encoding a WD40 repeat protein is involved in anthocyanin biosynthesis and trichome development (Walker *et al.*, 1999). It has been clarified that TTG1 interacts with different R2R3-MYBs and bHLHs to form different MBW complexes, which play roles in anthocyanin accumulation in vegetative tissues or proanthocyanidin biosynthesis in developing seeds (Xu *et al.*, 2014). *PAC1*, the homologous gene of *TTG1* in *Zea mays*, functionally complement the *ttg1* mutant indicating the conserved function of these WD40 proteins between dicots and monocots (Carey *et al.*, 2004). However, little information is known about WD40-encoding genes involved in anthocyanin biosynthesis in rice. It is predicted that the protein sequence of *Os02g0682500* is closely related to that of *TTG1* and *PAC1*. Nonetheless, there is no nucleotide difference in the coding and regulatory region of *Os02g0682500* between PH NIL and Nipponbare. Further analysis of the natural variations of this gene in rice germplasm revealed no functional mutations occurred (data not shown). This indicates that *Os02g0682500* may not be essential for anthocyanin biosynthesis in rice. It could also be that this gene plays an important role in rice growth and any functional mutations in this gene may have a lethal effect. Besides, it also should be noted that the anthocyanin-related WD40 proteins are functionally redundant in rice. More work is needed to clarify this.

### Conserved regulation patterns of flavonoid biosynthesis in plants

In maize, it was clearly verified that the R3 repeat of ZmC1 (the homolog of C1) interacts with the N-terminal region of ZmB/R (S1 homologs in maize) (Goff *et al.*, 1992; Grotewold and Chandler, 2000). The combined transcript complex activates expression of *ZmA1* (*A1* homolog in maize) by either protein directly binding to a specific DNA sequence in the *ZmA1* promoter. The DNA binding motifs of C1 and S1 in the promoter of *A1* are the same as that in the maize *ZmA1* promoter (see Supplementary Fig. S5C; Sainz *et al.*, 1997; Kong *et al.*, 2012). All these results indicate the conserved regulatory pattern of flavonoid biosynthesis in the grass family.

By homologous comparisons of C1 protein sequences between *Oryza sativa*, *Zea mays*, and Arabidopsis, we found that this MYB transcription factor in the three species had higher similarity in the R2 and R3 domains (86%) when compared to other regions (31%) (data not shown). Likewise, S1, ZmB/R, and TT8, as homologs in the three species, had a higher similarity in their MYC N-terminal regions (74%) than their C-terminal regions (data not shown). We consider that these conserved domains are fundamental in maintaining their functions in regulating flavonoid biosynthesis in plants.

### Multi-alleles of *S1* correlate with specific tissue pigmentation in rice

In wild rice, purple color occurs in various organs, but not in the hull and pericarp (Li and Chen, 1993). The pigmentation pattern in cultivars is more diverse as the pericarp and hull can also be colored purple.

In the present study, we confirmed that *S1* acted as a hull-specific pigmentation gene, but the functional nucleotides were not identified. We found that the promoter sequence of *S1* in the PH NIL was the same as that in Nipponbare, but different changes may occur in the 3′ end of *S1* in PH NIL. We failed to amplify products from the seventh exon to the 3′-UTR in PH NIL. However, amplification succeeded in PA NIL and its sequence was same as that in Nipponbare. But further investigation of the *S1* sequence in 13 varieties with purple hull revealed that the 3′-end ‘black hole’ was not universal (see Supplementary Fig. S14). We speculate that other structural variations in *S1* rather than mutations in translated products have a determinant effect on the acquisition of pigmentation in the hull.

## Supplementary data

Supplementary data are available at JXB online.

Fig. S1. Color phenotypes in rice floral organs.

Fig. S2. Plasmid constructs for the transient expression assay.

Fig. S3. Mapping of *C1*.

Fig. S4. Mapping of *S1*.

Fig. S5. Mapping of *A1*.

Fig. S6. Expression of *C1* in overexpressed transgenic lines.

Fig. S7. Expression of *C1* and *A1* in *C1* RNAi lines.

Fig. S8. Overexpression of *S1* in PA NIL.

Fig. S9. Principal component analysis of the LC-MS data.

Fig. S10. Haplotype analyses of *C1* and *A1*.

Fig. S11. Proposed evolutionary pathway of *Oryza sativa.*

Fig. S12. Proposed model of *C–S–A* gene system manipulating rice coloration.

Fig. S13. Overexpression of *S1* in NIP.

Fig. S14. Investigation of the 3′ end structure of *S1* in 13 purple hulled accessions by PCR.

Table S1. Information on rice varieties used in this study.

Table S2. Primers used for fine mapping, gene sequencing, RT-PCR, and vector construction.

Table S3. Summary of the *C1* and *A1* haplotypes in accessions from the rice mini core collection.

Table S4. Summary of phenotypic data of segregating lines.

Table S5. Data matrix of 161 flavonoid metabolites detected in rice hulls.

Table S6. Data sets of differentiated metabolites between pairwise comparisons of three groups.

Table S7. Forty-nine flavonoid metabolites with differences among lines with purple, brown, and straw-white hulls.

Table S8. Major flavonoid compounds identified in purple and brown hulls.

Table S9. Natural variations in CDS regions of five structural genes.

Table S10. Nucleotide variation and neutrality test of *C1*.

Table S11. Nucleotide variation and neutrality test of *A1*.

Table S12. Seven rice varieties with purple pericarp in the mini core collection.

Supplementary Figures and Tables

Supplementary Table 1

Supplementary Table 2

Supplementary Table 3

Supplementary Table 5

Supplementary Table 6

Supplementary Table 7
