# Supplementary material for: The C–S–A gene system regulates hull pigmentation and reveals evolution of anthocyanin biosynthesis pathway in rice
Source: J Exp Bot. 2018 Jan 18;69(7):1485–98. doi: 10.1093/jxb/ery001 (PMC5888925; doi:10.1093/jxb/ery001)
Supplement: Supplementary Figures and Tables [file ery001_suppl_figs_tabs_s1.pdf]

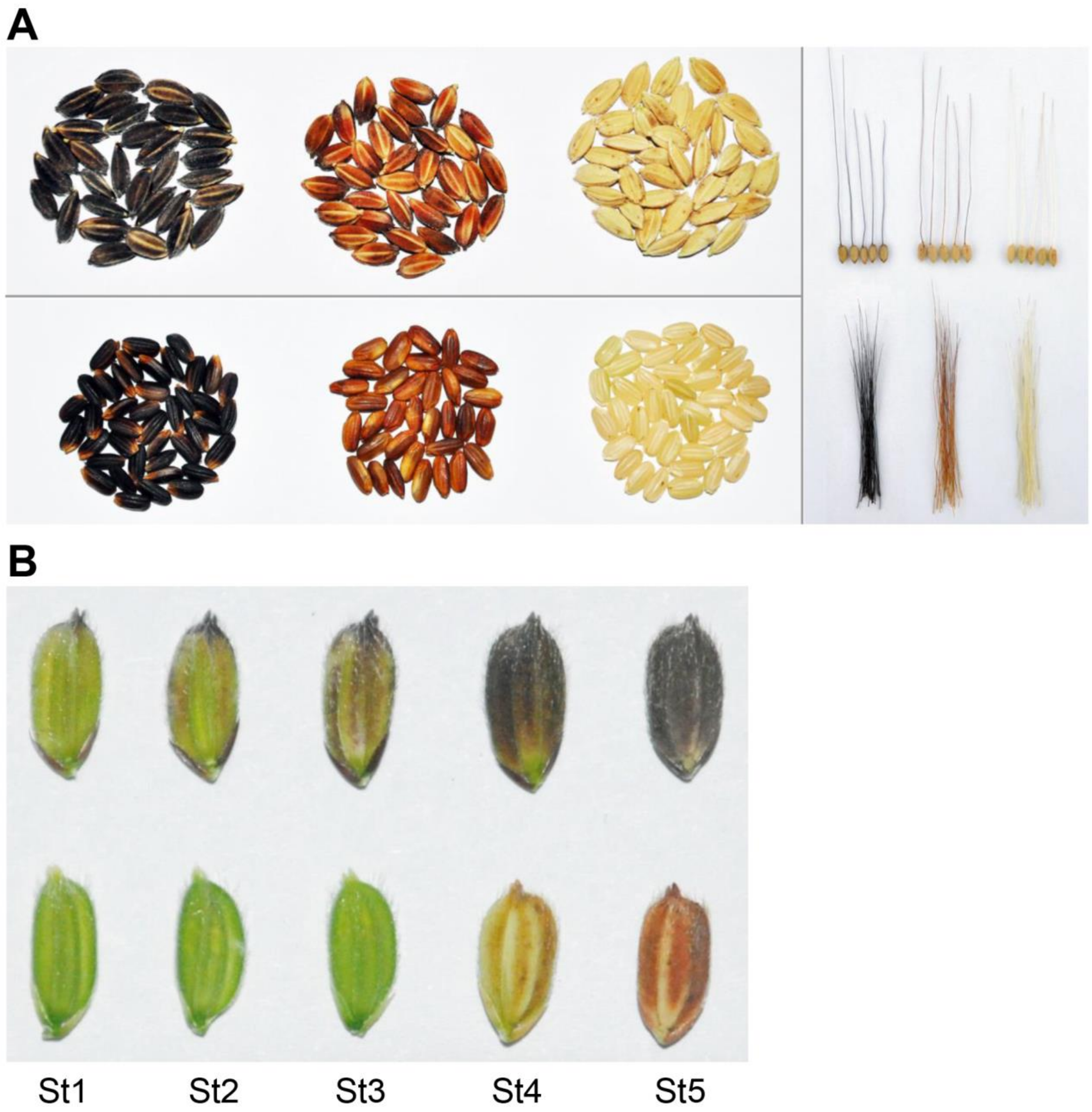

Fig. S1. Color phenotypes in rice floral organs.

(A) Purple, brown and straw-white colors in rice hulls (uper left), pericarp (lower left) and awn (right). Bottom right shows awns cut from uper spikelets. (B) Development of purple and brown color in rice hulls during progressive reproductive stages. St1, initial heading stage; St2, flowering stage; St3, filling stage; St4, wax ripeness stage; St5, fully ripened stage.

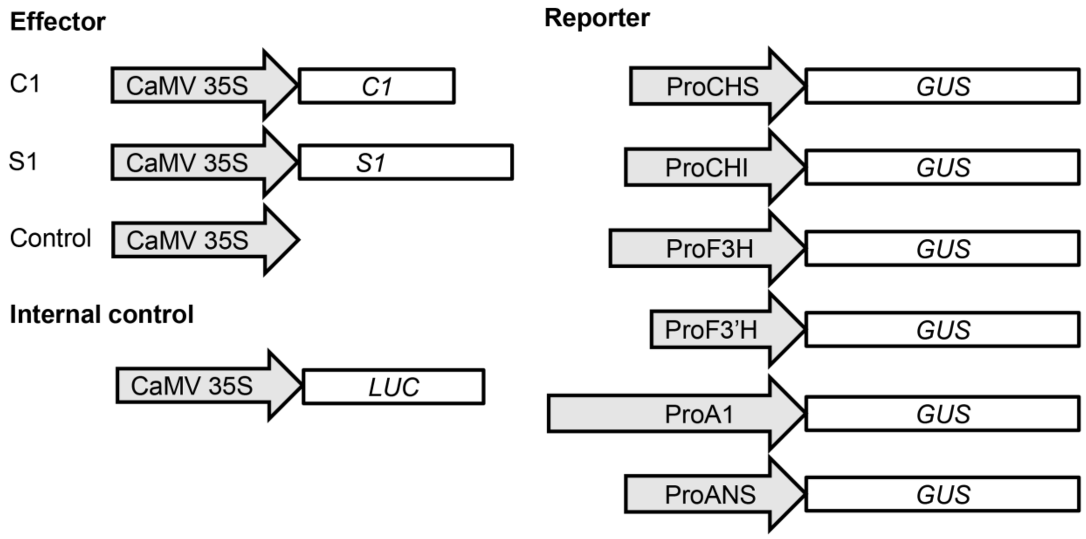

Fig. S2. Plasmid constructs for the transient expression assay. The CDS of *C1* and *S1* were driven by the CaMV35S promoter.

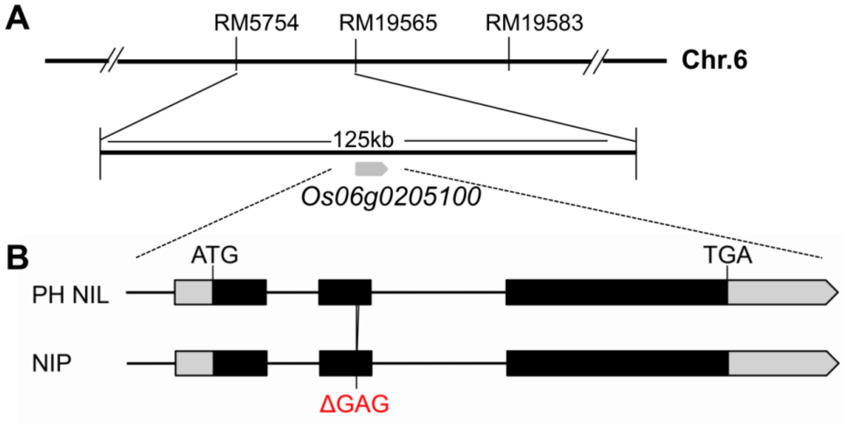

Fig. S3. Mapping of *C1*. (A) Fine mapping of *C1*. (B) The structure of *C1* in PH NIL and Nipponbare (NIP). Black boxes indicate exons. 'ΔGAG' is the deletion in the second exon of NIP.

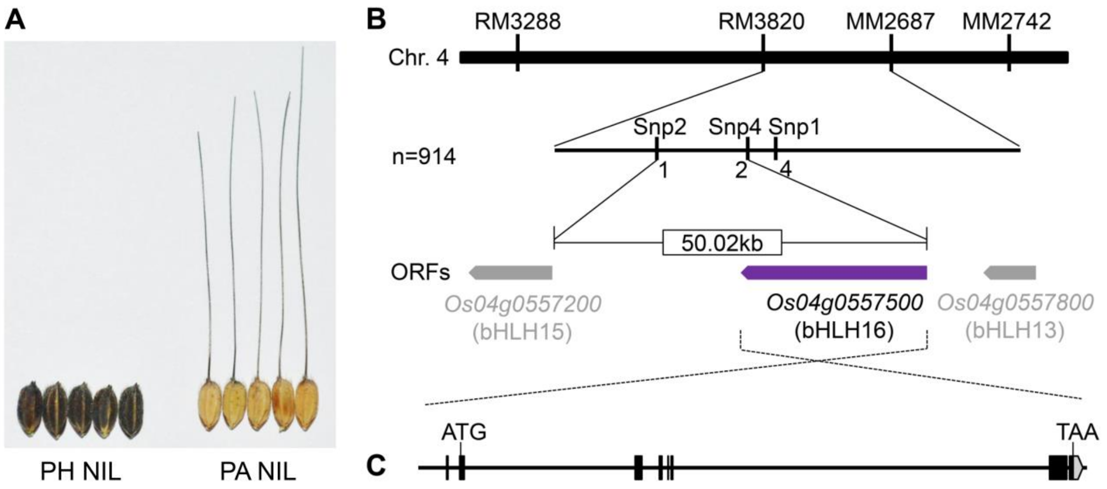

Fig. S4. Mapping of *S1*. (A) Hull color phenotypes of two parents, PH NIL and PA NIL. (B) Fine mapping of *S1*. (C) *S1* gene structure.

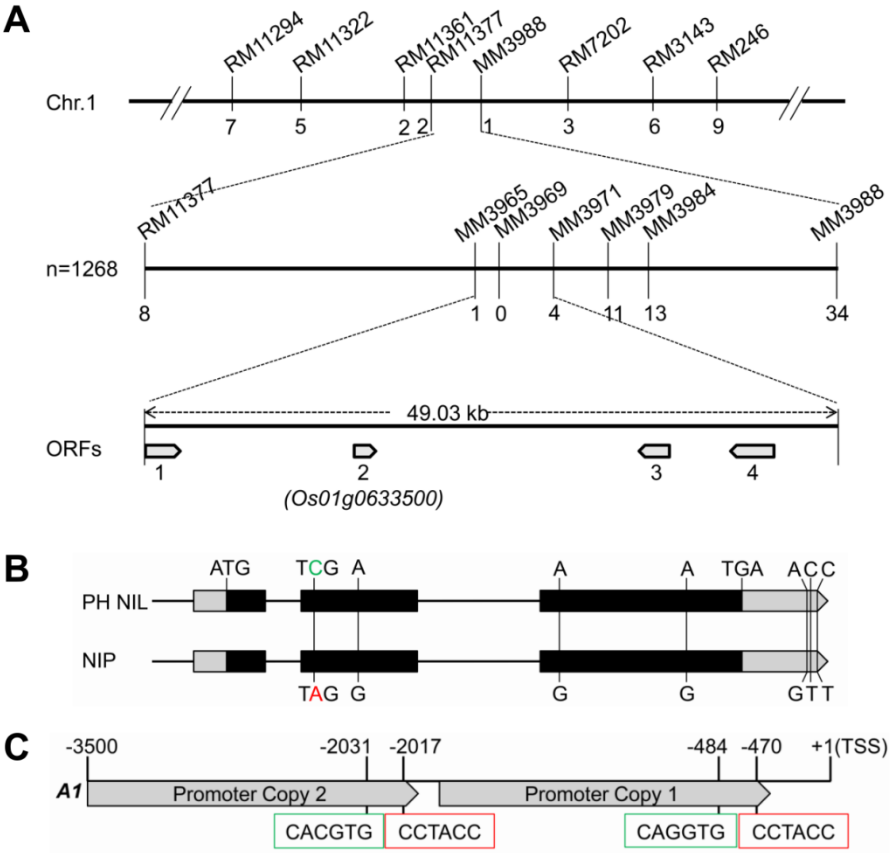

Fig. S5. Mapping of *A1*. (A) Fine mapping of *A1*. (B) Schematic of the gene structure and allelic variations in *A1* between PH NIL and NIP. The casual SNP is marked as green or red colored letters. (C) Schematic of the *A1* promoter. Two copies were in the promoter. The sequence circled by red boxes indicate the C1 binding motifs, whereas green boxes indicate the S1 binding motifs.

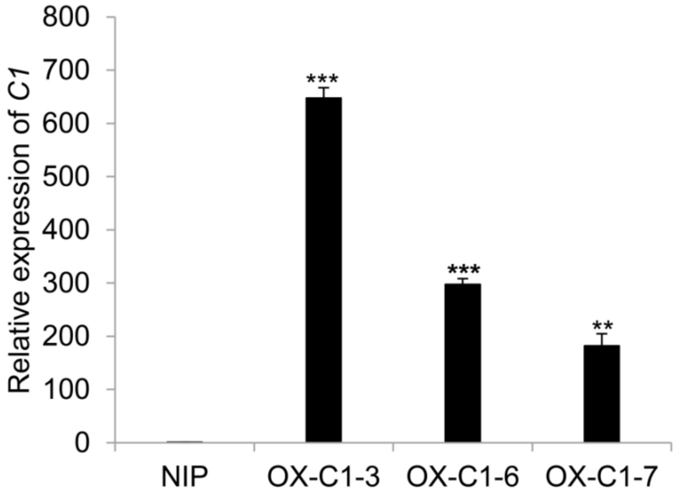

Fig. S6. Expression of *C1* in over-expressed transgenic lines. Three independently over-expressed transgenic lines, OX-*C1*-3, OX-*C1*-6 and OX-*C1*-7 were assayed for *C1* expression. Error bars represented ±SD (n=3). Asterisks indicate significant differences between NIP and transgenic lines at ***P* < 0.01 and ****P* < 0.001 (Student's *t*-test), respectively.

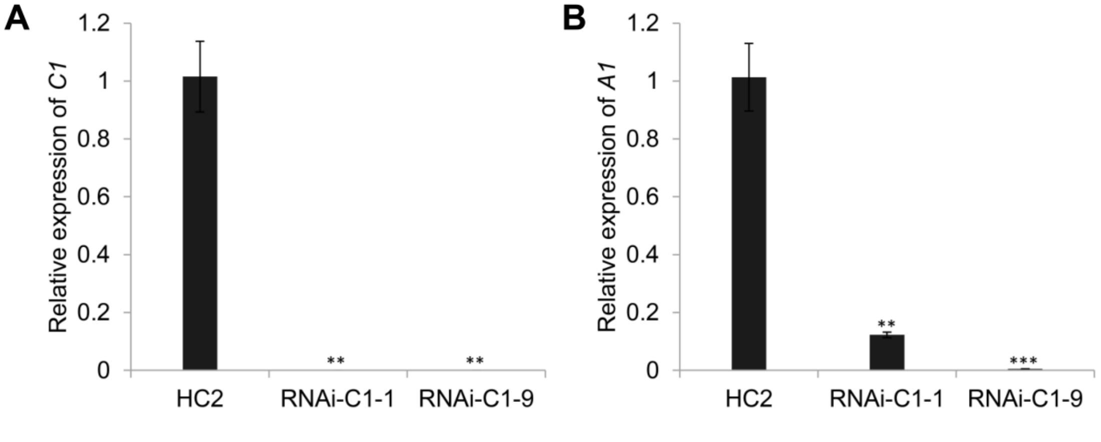

Fig. S7. Expression of *C1* and *A1* in *C1* RNAi lines. (A and B) The expression of *C1* and *A1* in transgenic lines, respectively. Error bars represented ±SD (n=3). Asterisks indicate significant differences between PH NIL and transgenic lines at ***P* < 0.01 and ****P* < 0.001 (Student's *t*-test), respectively.

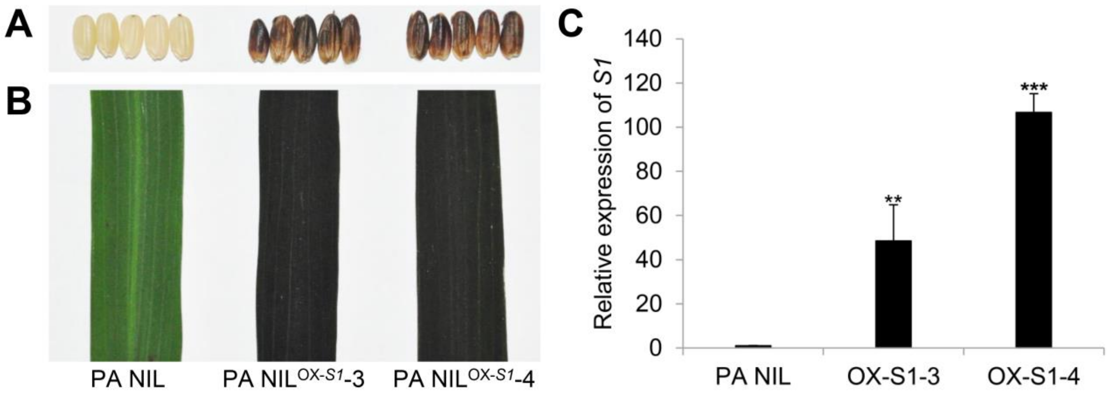

Fig. S8. Over-expression of *S1* in PA NIL. (A and B) Pericarp and leaf blade colors of over-expression transgenic lines in PA NIL background, respectively. (C) Expression levels of *S1* in PA NIL and transgenic lines. Error bars represented ±SD (n=3). Asterisks indicate significant differences between PA NIL and transgenic lines at $^{**}P < 0.01$ and $^{***}P < 0.001$ (Student's *t*-test), respectively.

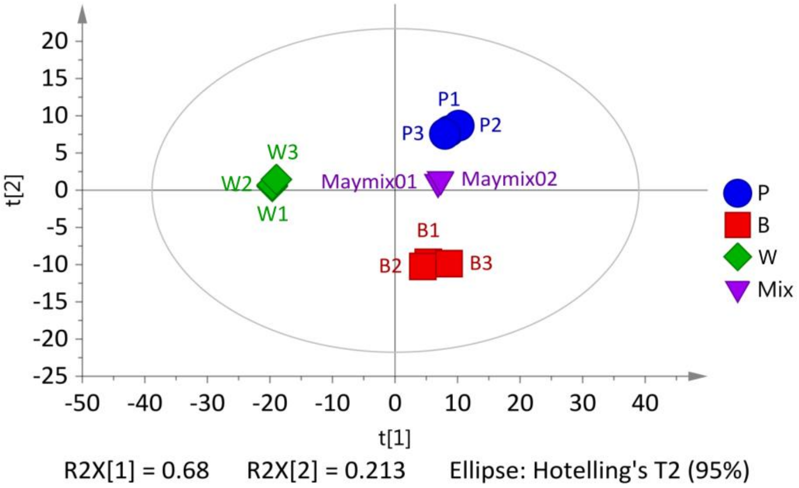

Fig. S9. Principal component analysis of the LC-MS data. Differences between metabolic profiles of purple, brown and straw-white hulls were assessed by PCA of LC-MS data sets. 'P' represents purple hull, 'B' represents brown hull and 'W' represents straw-white hull, 'Mix' indicates the three kinds of colored hulls mixed equally. Each colored hull was assayed in three replications; mixtures were detected and assayed twice.

A

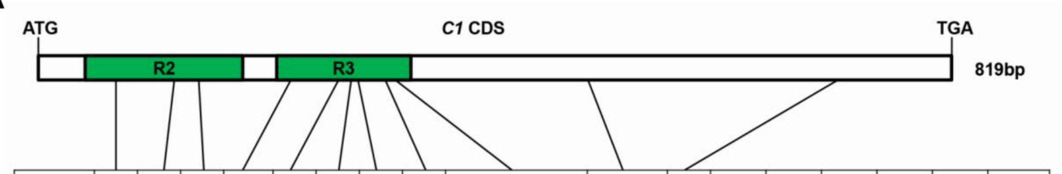

Position	70	122	143	226	269	281	287	311	320	493-537	716						
A.A. Sub	V24M	P41R	R48Q	In/Del E	S90F	G94D	L96P	F.S.Stop	F.S.Stop	F.S.Stop	A239V	*Aus*	*Ind*	*Aro*	*J-te*	*J-tr*	No. of accessions
hap1	G	C	G	GAG	C	G	T	TC	ACTGGAACAG	-	C		3	4	38	7	52
hap2	G	C	G	GAG	C	G	T	TC	ACTGGAACAG	-	T		19		1		20
hap3	G	C	A	GAG	C	G	T	TC	ACTGGAACAG	-	C				1		1
hap4	A	C	G	GAG	C	G	T	TC	ACTGGAACAG	-	C					1	1
hap5	G	C	G	---	C	G	T	TC	ACTGGAACAG	-	C				2		2
hap6	G	C	G	GAG	C	G	T	--	ACTGGAACAG	-	C		1		3		4
hap7	G	C	G	GAG	C	G	T	TC	----------	-	C	1	37	2	2	3	45
hap8	G	G	G	GAG	C	G	T	TC	----------	-	C	1					1
hap9	G	C	G	GAG	C	G	T	TC	ACTGGAACAG	Sub1	C		1		9		10
hap10	G	G	G	GAG	C	G	T	TC	ACTGGAACAG	-	C	2	2				4
hap11	G	G	G	GAG	C	A	T	TC	ACTGGAACAG	-	C			1			1
hap12	G	C	G	GAG	T	G	T	TC	ACTGGAACAG	-	C				2		2
hap13	G	C	G	GAG	C	G	C	TC	ACTGGAACAG	-	C		2				2

B

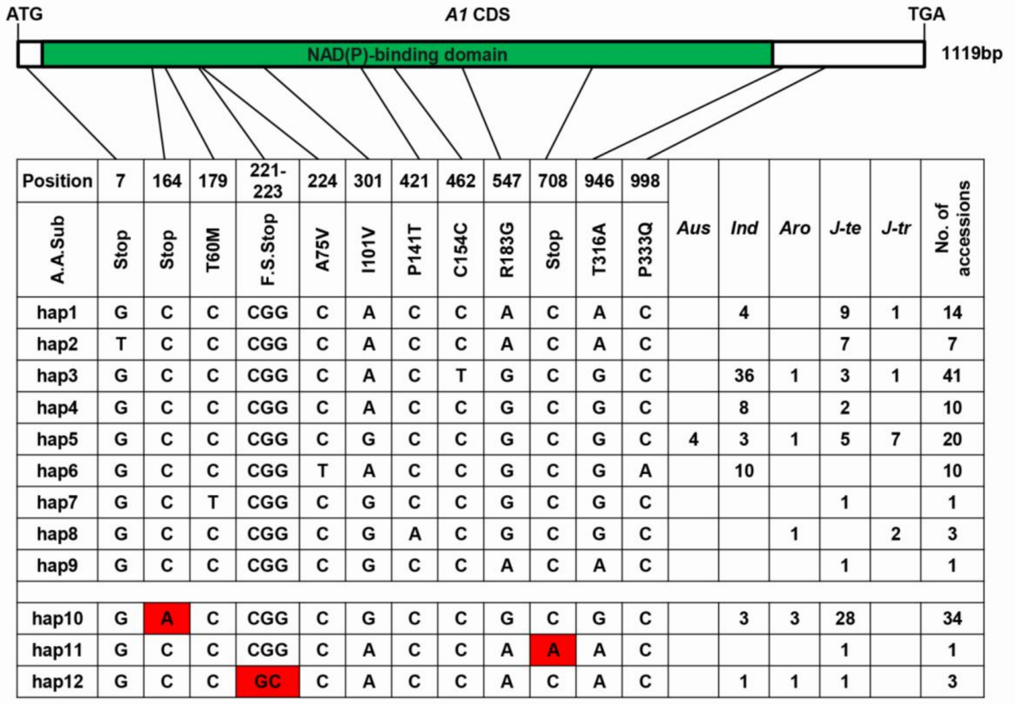

Position	7	164	179	221-223	224	301	421	462	547	708	946	998						
A.A.Sub	Stop	Stop	T60M	F.S.Stop	A75V	I101V	P141T	C154C	R183G	Stop	T316A	P333Q	*Aus*	*Ind*	*Aro*	*J-te*	*J-tr*	No. of accessions
hap1	G	C	C	CGG	C	A	C	C	A	C	A	C		4		9	1	14
hap2	T	C	C	CGG	C	A	C	C	A	C	A	C				7		7
hap3	G	C	C	CGG	C	A	C	T	G	C	G	C		36	1	3	1	41
hap4	G	C	C	CGG	C	A	C	C	G	C	G	C		8		2		10
hap5	G	C	C	CGG	C	G	C	C	G	C	G	C	4	3	1	5	7	20
hap6	G	C	C	CGG	T	A	C	C	G	C	G	A		10				10
hap7	G	C	T	CGG	C	G	C	C	G	C	G	C				1		1
hap8	G	C	C	CGG	C	G	A	C	G	C	G	C			1		2	3
hap9	G	C	C	CGG	C	G	C	C	A	C	A	C				1		1
hap10	G	A	C	CGG	C	G	C	C	G	C	G	C		3	3	28		34
hap11	G	C	C	CGG	C	A	C	C	A	A	A	C				1		1
hap12	G	C	C	GC	C	A	C	C	A	C	A	C		1	1	1		3

Fig. S10. Haplotype analyses of *C1* and *A1*. (A) *C1* nucleotide sequences of accessions in the mini core collection were compared with PH NIL (hap1). The number of accessions with each haplotype (hap1-13) is shown in the right column. Polymorphic nucleotides causing loss-of-function are in red; deletion and insertion sites are indicated by dashed lines; 'Sub1' is the substitution of

‘ACGGCAGCGGCGGCGGCGGCGGCGACGACGACCACCGTGTGGGCG’ to ‘GCAGCCAGCCT’. F.S., frame shift. (B) *A1* nucleotide sequences of accessions in the mini core collection were compared with PH NIL (hap1). Twelve haplotypes were defined; polymorphic nucleotides for loss-of-function types are in red.

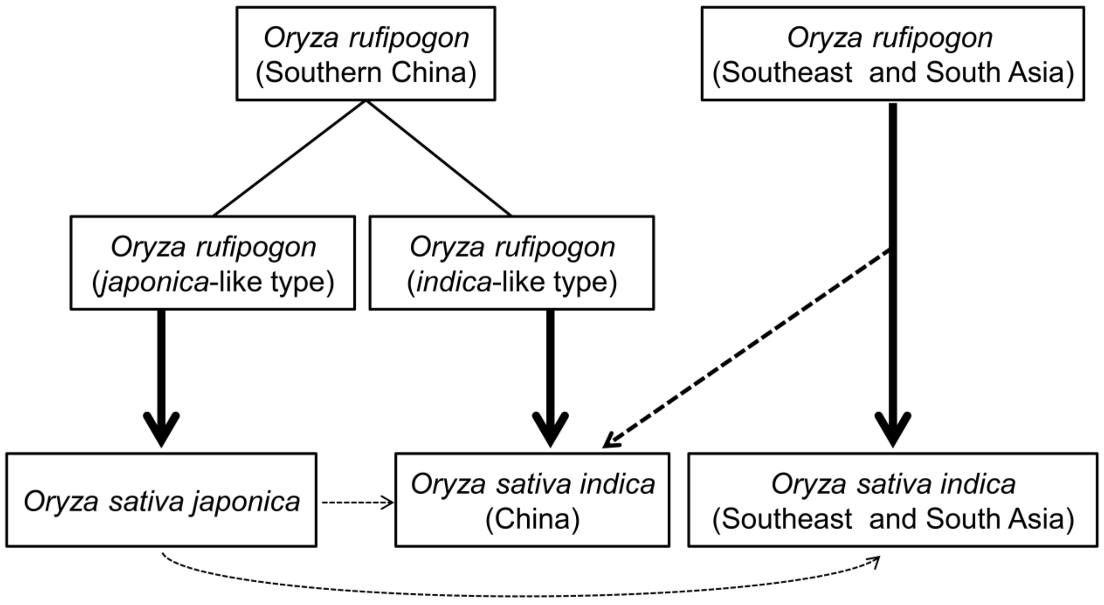

Fig. S11. Proposed evolution pathway of *Oryza sativa*. Arrows indicate the origins of cultivar from their recent ancestors.

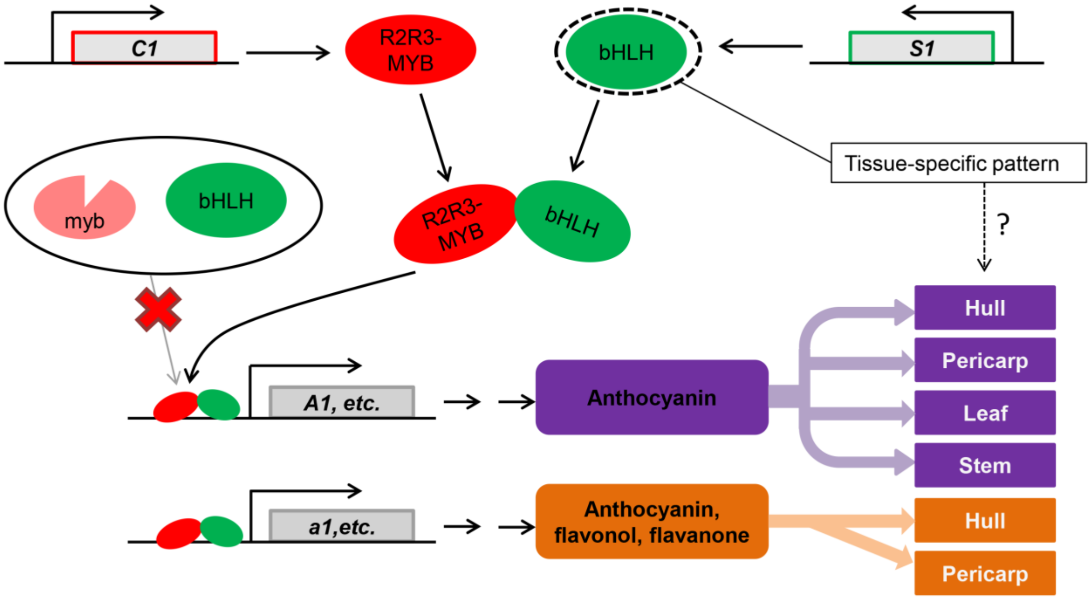

Fig. S12. Proposed model of *C-S-A* gene system manipulating rice coloration. The coloration is initiated by transcription factors encoded by *C1* and *S1*. The two proteins form the regulation complex and activate expression of *A1* and other structural genes. If *C1* losses its function, the two proteins cannot interact to form the complex and therefore cannot activate the down-stream genes expression. The enzymes translated by highly expressed structural genes catalyze the substrates conversion to flavonoid end-products. During flavonoid production, the DFR protein encoded by *A1* plays an important role in the anthocyanin biosynthesis branch pathway. When the DFR changes or weakens in catalytic activity, the flavonoid metabolites convert to the flavonol and flavanone pathway. *S1* determines pigmentation specificity in various rice tissues.

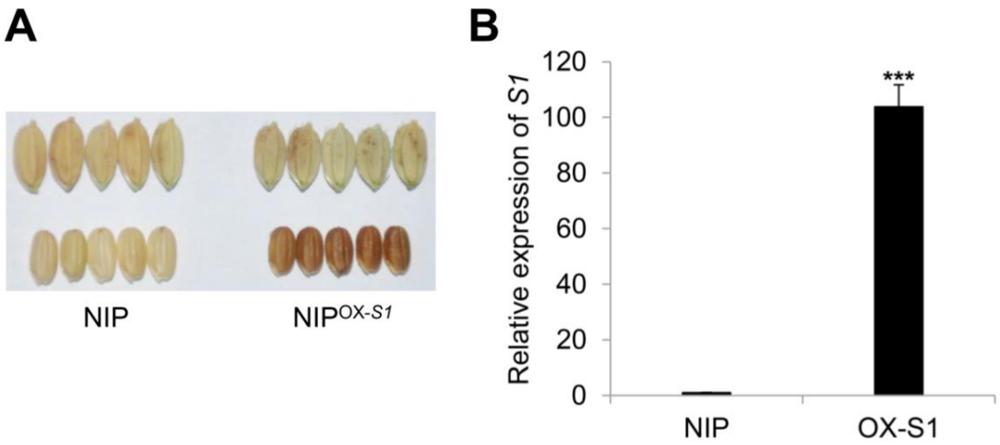

Fig. S13. Over-expression of *S1* in NIP. (A) Hull (upper) and pericarp (lower) colors of NIP and transgenic lines. (B) Expression of *S1* in parent and transgenic line. Error bars represented ±SD (n=3). Asterisks indicate significant differences between NIP and transgenic lines at ***$P < 0.001$ (Student's *t*-test).

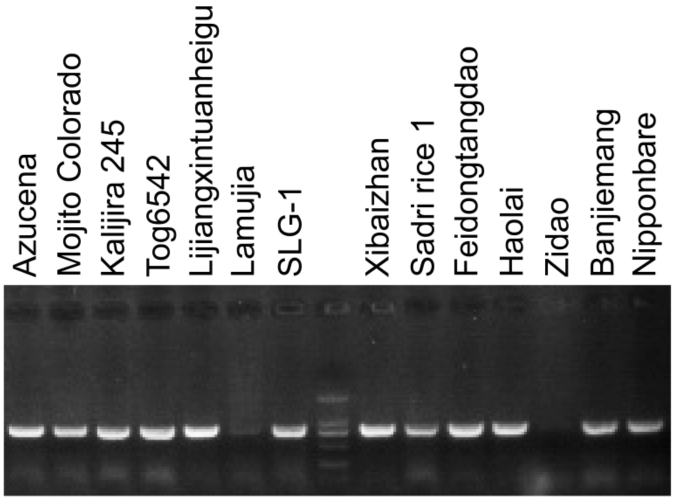

Fig. S14. Investigation of the 3' end structure of *S1* in 13 purple hulled accessions by PCR. PCR products were amplified using the primer pairs S1ex7-8F/R. Only two accessions failed to amplify.

Table S1. Information on rice varieties used in this study.

Table S2. Primers used for fine mapping, gene sequencing, RT-PCR, and vector construction.

Table S3. Summary of the *C1* and *A1* haplotypes in accessions from the rice mini core collection.

Table S4. Summary of phenotypic data of segregating lines.

Gene-ration	Serial no.	Purple hull	Brown hull	Purple apiculus	Brown apiculus	Straw -white	Total no.	Segregation ratio	*P* value
F_3	a	152	47	-	-	66	265	9:3:4	0.901
	b	91	-	30	-	49	170	9:3:4	0.515
F_4	Ⅰ-1	67	-	-	-	22	89	3:1	0.951
	Ⅰ-2	-	62	-	-	25	87	3:1	0.421
	Ⅰ-3	62	-	-	-	23	85	3:1	0.661
	Ⅰ-4	-	-	53	-	17	70	3:1	0.890
	Ⅱ	49	-	11	-	-	60	3:1	0.233
	Ⅲ	68	25	-	-	-	93	3:1	0.631
F_3(HHH type)		150	47	38	15	89	339	27:9:9:3:16	0.630

* ‘HHH type’ means F_2 individuals with heterozygous genotypes at all three loci.

Table S5. Data matrix of 161 flavonoid metabolites detected in rice hulls.

Table S6. Data sets of differentiated metabolites between pairwise comparisons of three groups.

Table S7. Forty-nine flavonoid metabolites with differences among lines with purple, brown and straw-white hulls.

Table S8. Major flavonoid compounds identified in purple and brown hulls.

	No.	Name	Flavonoid	Purple/ Brown	Purple/ Straw-white	Brown/ Straw-white
I	RF098	Naringenin 7-O-glucoside	Flavanone	5.1	**27.9**	5.5
	RF040	Luteolin O-rutinoside	Flavone	13.9	**81.9**	5.9
	RF009	Apigenin-7-O-glucoside	Flavone	574.1	**829.8**	1.4
	RF016	C-hexosyl-luteolin O-hexoside	Flavone	2.6	**30.7**	11.7
	RF036	C-hexosyl-chrysoeriol O-hexoside	Flavone	2.8	**23.8**	8.4
	RF011	Catechin	Proanthocyanidin	24.0	**37.7**	1.6
II	RF057	Hesperetin	Flavanone	0.3	24.2	**77.9**
	RF050	Hesperetin 5-O-glucoside	Flavanone	0.2	236.7	**1089.2**
	RF041	Quercetin	Flavonol	0.1	25.7	**216.2**
	RF043	Rutin	Flavonol	0.2	207.7	**1337.2**
	RF042	Delphinidin 3-O-rutinoside	Anthocyanin	0.3	167.8	**505.3**

* The flavonoid compounds with content fold changes more than 20 are listed in comparison between purple and straw-white groups, brown and straw-white groups. The highest values are in bold.

Table S9. Natural variations in CDS regions of 5 structural genes.

Gene name	Gene locus	Nonsense mutations	Indels	Nonsynonymous SNPs
CHS	*Os11g0530600*	0	0	1
CHI	*Os03g0819600*	0	0	2
F3H	*Os04g0662600*	0	0	2
F3'H	*Os10g0320100*	0	0	4
ANS	*Os01g0372500*	0	1	16

Table S10. Nucleotide variation and neutrality test of *C1*.

Taxon	N	S	H	π_T	θ_T	π_{sil}	θ_{sil}	Tajima's *D*	Fu Li's *D*
Wild rice	108	26	21	0.00121	0.00112	0.00147	0.00137	0.21310	1.12705
O.sativa	363	44	10	0.00189	0.00155	0.00191	0.00155	0.62207	2.30481**
ssp. japonica	135	44	7	0.00045	0.00183	0.00018	0.00183	-2.30241**	-5.37220**
ssp. indica	228	42	8	0.00247	0.00159	0.00276	0.00167	1.60459	1.59793
Total colored	131	38	6	0.00323	0.00159	0.00389	0.00184	3.12887**	2.10205**
Total straw-white	232	44	7	0.00065	0.00167	0.00038	0.00166	-1.76501*	2.26598**

N, total number of accession sequences; S, number of polymorphic (segregating) sites; H, number of haplotypes; π_T, average number of nucleotide differences per site calculated based on the total number of polymorphic sites; θ_T, Watterson estimator of θ per base pair calculated based on the total number of polymorphic sites; π_{sil}, average number of pairwise nucleotide differences per site calculated based on silent sites; θ_{sil}, Watterson estimator of θ per base pair calculated based on silent sites.*P <0.05,**P <0.01.

Table S11. Nucleotide variation and neutrality test of *A1*.

Taxon	N	S	H	π_T	θ_T	π_{sil}	θ_{sil}	Tajima's *D*	Fu Li's *D*
Wild rice	108	100	39	0.00137	0.00176	0.00140	0.00183	-0.72888	0.91872
O.sativa	363	108	15	0.00268	0.00155	0.00271	0.00155	2.19123*	2.93988**
ssp. japonica	135	108	8	0.00365	0.00183	0.00367	0.00183	3.20637**	2.56895**
purple	36	104	5	0.00414	0.00232	0.00428	0.00241	2.91145**	1.83070**
brown	30	98	3	0.00116	0.00229	0.00116	0.00227	-1.89030*	1.76030**
ssp. indica	228	108	12	0.00063	0.00167	0.00064	0.00167	-1.91709*	2.47418**

* See footnote to Table S8 for abbreviations.

Table S12. Seven rice varieties with purple pericarp in the mini core collection.

Variety Name	Subgroup	Origin country	*C1*	*A1*	Pericarp color	Hull colr
Heizhandao	*J-Te*	China	hap1	hap3	Purple	Purple
Lijiangxiaoheigu	*J-Te*	China	hap1	hap4	Purple	Purple
Hei Mi Chan	*Ind*	China	hap2	hap3	Purple	Purple
Zimi	*Ind*	China	hap7	hap3	Purple	Straw-white
Zaoshuxianghei	*Ind*	China	hap7	hap3	Purple	Straw-white
PPR1	*J-Te*	China	hap9	hap3	Purple	Straw-white
PPR2	*Ind*	China	hap7	hap3	Purple	Straw-white

* Functional and non-functional allelic of *C1* and *A1* are highlighted with green and red, respectively.

Table S1, S2, S3, S5, S6 and **S7** were prepared separately as excel documents.
